# The Role of Androgen Receptor and microRNA Interactions in Androgen-Dependent Diseases

**DOI:** 10.3390/ijms23031553

**Published:** 2022-01-28

**Authors:** Agnieszka Bielska, Anna Skwarska, Adam Kretowski, Magdalena Niemira

**Affiliations:** 1 Clinical Research Centre, Medical University of Bialystok, 15-276 Bialystok, Poland; adamkretowski@wp.pl; 2 Department of Leukemia, Anderson Cancer Center, The University of Texas M.D., Houston, TX 77030, USA; askwarska@mdanderson.org; 3 Department of Endocrinology, Diabetology and Internal Medicine, Medical University of Bialystok, 15-276 Bialystok, Poland

**Keywords:** microRNA, androgen receptor, cancer, prostate cancer, breast cancer

## Abstract

The androgen receptor (AR) is a member of the steroid hormone receptor family of nuclear transcription factors. It is present in the primary/secondary sexual organs, kidneys, skeletal muscles, adrenal glands, skin, nervous system, and breast. Abnormal AR functioning has been identified in numerous diseases, specifically in prostate cancer (PCa). Interestingly, recent studies have indicated a relationship between the AR and microRNA (miRNA) crosstalk and cancer progression. MiRNAs are small, endogenous, non-coding molecules that are involved in crucial cellular processes, such as proliferation, apoptosis, or differentiation. On the one hand, AR may be responsible for the downregulation or upregulation of specific miRNA, while on the other hand, AR is often a target of miRNAs due to their regulatory function on *AR* gene expression. A deeper understanding of the AR–miRNA interactions may contribute to the development of better diagnostic tools as well as to providing new therapeutic approaches. While most studies usually focus on the role of miRNAs and AR in PCa, in this review, we go beyond PCa and provide insight into the most recent discoveries about the interplay between AR and miRNAs, as well as about other AR-associated and AR-independent diseases.

## 1. Introduction

The androgen receptor (AR), together with the estrogen, progesterone, and glucocorticoid receptors, belongs to the steroid hormone receptor family, which acts as a ligand-dependent transcription factor. The *AR* gene is a single gene that is ∼90 kbp in size and located on the X-chromosome at Xq11–12 [1]. The AR is mainly present in the primary/secondary sexual organs, but is also present in the kidneys, skeletal muscles, adrenal glands, skin, nervous system, and breast [2,3,4]. The AR ligands, testosterone and its metabolite 5α-dihydrotestosterone, are the most active androgen hormones and are responsible for various physiological effects on the reproductive and non-reproductive systems [5]. The circulating androgens bind to the androgen receptor that is located in the cytoplasm, which is associated with heat shock proteins (HSP) and other chaperons, which, in turn, initiates the transport of the AR dimers to the nucleus. There, AR activates or represses its respective target genes, regulating mRNA expression [6,7]. Abnormal AR functioning has been identified in numerous diseases, such as prostatic hyperplasia, prostate cancer, androgen insensitivity syndrome, hypogonadism, or spinal bulbar muscular atrophy [8]. It is therefore of critical importance to understand the molecular mechanisms governing AR activity and regulation in these pathological states. Particularly, in many AR-associated disease entities, the role of AR and its interaction with microRNA (miRNA) is still poorly described and is rarely investigated. However, most recent studies provide new evidence for AR–miRNA crosstalk, even in diseases such as lung cancer, liver cancer, and renal cancer, among others. In this review, we focused on the role of AR–miRNA interactions in the progression of various diseases.

## 2. miRNA

MiRNAs are small (about 17–25 nucleotides in length), non-coding, single-stranded, endogenous molecules that play an important role in the regulation of post-transcriptional gene expression by interacting with the 3′ untranslated region (3′UTR) of its target messenger RNA (mRNA) [9,10]. The complementary degree between the miRNA sequence and its target mRNA determines the regulatory effect of miRNA [11]. The association of miRNA with its target mRNA can result in mRNA cleavage, translational repression, or mRNA deadenylation [12,13]. In rare cases, miRNA can activate mRNA translation and can increase target protein levels [14]. A single miRNA can target and regulate several different genes, and a single gene can be regulated by many miRNAs, creating a complex network of interactions [15]. More than 50% of protein-coding genes are believed to be regulated by miRNAs [16]. MiRNAs play a fundamental role in a large number of key processes, such as development, proliferation, apoptosis, metabolism, differentiation, inflammation, metastasis, angiogenesis, and tumorigenesis [10,17,18,19]. By 2002, it had already been shown that miRNAs are involved in cancer [20]. In recent years, miRNAs have become useful biomarkers for the diagnosis, prognosis, and therapy strategies in not only many types of cancers, such as breast, prostate, and lung cancer or melanoma, but also in metabolic, cardiovascular, and neuronal diseases [21,22,23,24]. MiRNAs can play a role as oncogenes (oncomiRs) or as suppressors of oncogenic transformation [23]. OncomiRs are frequently upregulated in cancer, and can promote tumour development by inhibiting the tumour suppressor gene, hence stimulating tumorigenesis. In contrast, tumour suppressor miRNAs are frequently downregulated in cancer and act by inhibiting oncogenes, repressing tumour progression [25,26]. Therefore, the inhibition of oncomiRs and the overexpression of tumour suppressor miRNAs may be a very promising strategy for targeted cancer therapies.

**Figure 1:**
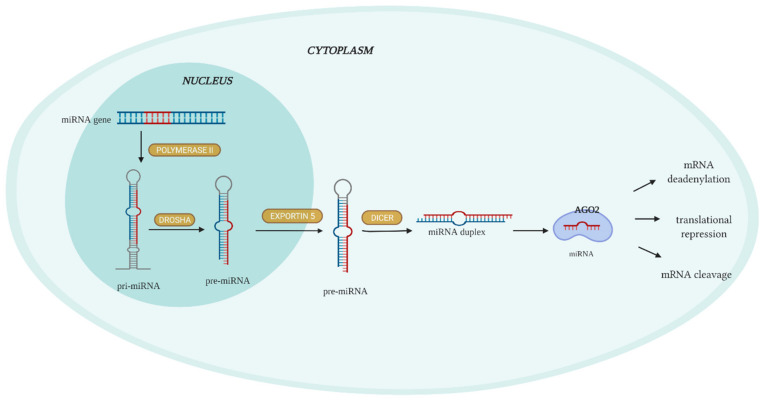
MicroRNA (miRNA) biosynthesis and functions. MiRNA gene is transcribed by polymerase II to primary RNA (pri-miRNA). The ribonuclease Drosha is involved in the process of changing pri-miRNA into pre-miRNA. Afterwards, pre-miRNA is transported via Exportin-5 from the nucleus to the cytoplasm. Dicer is an endonuclease that splits pre-miRNAs into short miRNA duplexes. The unknown helicase participates in the splitting of the miRNA duplexes. The mature miRNA binds to the Argonaute (Ago) protein, creating a complex that targets the 3′ UTR region of targeted mRNA. Illustration created using BioRender.com (access date: 28 December 2021).

Most miRNA genes are transcribed by RNA polymerase II in the nucleus as primary RNA (pri-miRNAs) (Figure 1). To form a precursor of miRNAs (pre-miRNAs), the stem–loop structure of a pri-miRNA is cleaved by the enzyme Drosha. The pre-miRNA, which is built from ∼70 nucleotides, is subsequently exported to the cytoplasm by the Exportin-5 protein. Once in the cytoplasm, the pre-miRNA is cleaved by the enzyme Dicer, which splits it into double-stranded miRNA. Finally, one of the strands of miRNA is removed, and another is bound to the AGO2 protein, which is a member of RISC (RNA-included silencing complex). This complex can target the 3′ UTR region of the mRNA, which results in mRNA deadenylation, translational repression, or mRNA cleavage [25,27,28].

## 3. miRNA and AR

**Figure 2:**
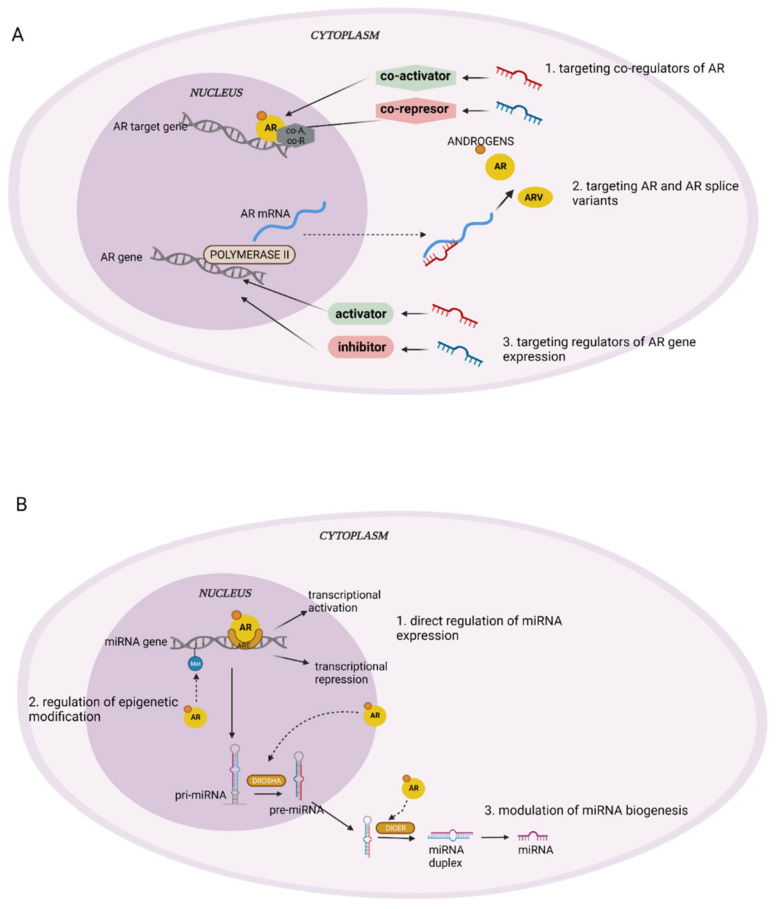
Main mechanisms of AR regulation by miRNA (**A**) and miRNA regulation by AR (**B**). Illustration created using BioRender.com (access date: 28 December 2021).

Most recent research emphasizes the importance of the interactions between miRNAs. AR expression can be regulated by different miRNAs directly or indirectly by affecting the expression of co-regulators (co-activators and co-repressors), which can shape AR functions [29,30,31,32] (Figure 2).

For example, prohibitin is considered to be a corepressor of AR. Fletcher et al. showed that prohibitin is targeted by miR-27a, which leads to the increased expression of AR target genes and PCa cell growth [31]. Another study showed that miR-137 targets AR co-activators, including an extended network of transcriptional coregulators, such as Lysine Demethylase 1A (*KDM1A),* Mediator Complex Subunit 1 (*MED1*), or Nuclear Receptor Coactivator 2 (*TIF2*) [32]. In PCa cell lines, miR-17-5p indirectly modulates the transcriptional activity of AR by targeting the p300/CBP-associated factor (PCAF) [31]. In addition, miRNAs can target the factors that regulate AR gene expression. MiR-let-7c indirectly represses AR activity by targeting the oncogenic transcription factor c-Myc [33]. The second regulatory mechanism of AR expression involves the direct targeting of AR or the AR splice variants (ARVs) by miRNAs [34]. For example, miR488* directly affects AR signalling by targeting the 3′ UTR of AR and downregulates AR protein expression in PCa cells, which leads to apoptotic cell death [35].

On the other hand, under various conditions, the androgens and AR may be responsible for the downregulation or upregulation of specific miRNAs (Figure 2). AR can directly regulate miRNAs by binding to specific DNA sequences, termed androgen response elements (AREs), in the regulatory region of the target genes. AREs are dihexameric motifs that are located in the enhancers and promoters of their target genes. As a result, transcriptional activation or, rarely, repression occurs. Wang et al. showed that AR upregulates miR-4496 expression by directly binding to the AREs of the miR-4496 promoter. Subsequently, the miR-4496 decreases the expression of β-catenin by directly targeting the 3′ UTR of the β-catenin-mRNA [36]. Another way that miRNA can be regulated by AR is through the indirect regulation of epigenetic modifications. In PCa cells, AR associates with KDM1A (lysine-specific demethylase 1), which enables the removal of repressive methyl marks in AR-targeted genes [37]. This also modulates the methylation of the promoter elements of AR-upregulated miRNAs (miR-22 and miR-29a) [38]. Finally, AR may modulate the biogenesis of miRNA. It has been shown that in PCa cells, AR can regulate the expression of the crucial enzymes that are required for miRNA biogenesis, such as Dicer and Drosha [31,39].

## 4. miRNA and AR in Various Diseases

While AR is commonly associated with prostate cancer, recent studies indicate that not only AR, but also the AR–miRNA interactions, play a key role in cancers, such as liver cancer, genitourinary cancer, and pancreatic cancer, in other diseases, such as PCO and cardiovascular disorders, and in normal processes, such as adipogenesis and placenta development. Table 1 provides insight into the novel findings regarding the AR–miRNA crosstalk that takes place during these processes. 

Table 2 presents the main oncomiRs and tumour suppressor miRNAs connected with androgen receptors in the different types of cancer discussed in this paper.

### 4.1. AR and miRNA in Breast Cancer

In general, androgens are considered male hormones, but they are also present in females. Androgens play a crucial role in female development and physiology [90,91], and a vast majority of breast cancers (BC) are positive for AR [45,92,93]. An increasing number of studies indicate a link between miRNA and AR in BC development and metastasis [94,95]. In many cases of triple-negative breast cancer (TNBC), the tumour cells show AR expression. It is possible that AR promotes the progression of this type of cancer through controlling the expression of miRNAs that are crucial for BC development, such as miR-125b, miR-21, and let-7a. These miRNAs target particular mRNAs that affect the protein expression involved in BC development, such as the transmembrane glycoprotein cluster of differentiation 44 (CD44), estrogen receptor (ER), progesterone receptor (PR), and receptor tyrosine-protein kinase erbB-2 (HER-2) [96]. In a recent study, Kalinina et al. showed that the changes in expression levels of AR, miR-185, miR-205, and miR-21 vary in specific BC subtypes [40]. MiR-185 and miR-205 were previously described as miRNAs that are able to directly target AR expression [97,98]. Interestingly, AR regulates the transcription of miR-21, which promotes PCa growth [99]. Huang et al. also confirmed a link between a miR-185-5p and AR in clear cell renal cell carcinoma (ccRCC). AR elevates the expression of miR-185-5p, which suppresses VEGF-C and increases HIF2α/VEGF-A expression [53]. In contrast, in breast cancer, androgens have been reported to act as negative modulators of the miR-21, which results in a reduction in BC cells proliferation. In such cases, AR acts as a transcriptional repressor of miR-21 expression [41]. MiR-21 often plays the role of the main onco-miRNA in carcinogenesis, and its expression is consistently high in hormone-dependent cancers, including PCa and BC [100,101]. MiR-206 is also regulated by hormones in both BC and PCa, and may act as a tumour suppressor as well as an oncogene [102,103]. Ahram et al. showed that AR may regulate the extracellular release of metalloprotease-13 (MMP13) in the BC cell lines by controlling miRNA expression. In response to the AR agonist CI-4AS-1, the expression of miR-100 and miR-125 was significantly reduced in MDA-MB-453 breast cancer cells, leading to the increased expression of miR-100 and miR-125 target metalloprotease-13 (*MMP13*) [42]. MiR-328-3p can mediate the AR regulation of BC, and AR controls the expression of *CD44* via miRNA-dependent and independent mechanisms in BC cells. In TNBC, exposure to DHT significantly upregulated the miR-328-3p level, leading to decreased expression of the miR-328-3p target *CD44* and the subsequent reduction in cell adhesion and migration [43]. In contrast, Al-Momany et al. recently showed that DHT was able to regulate the chemo-response in TNBC through a mechanism that was independent of miR-328-3p and ABCG2 [44]. Studies report that miR-9-5p acts as both an onco-miR and a tumour suppressor, and its role is still debated. However, a recent work by Li et al. suggested that the miR-9 level, and its role in BC, depends on the stage of the disease. MiR-9 may inhibit the occurrence of BC in the early stages of the disease, and may also act as an onco-miR in metastatic BC with a higher malignancy [104]. Bandini et al. suggested that miR-9-5p potentially targets *AR* and that it may play a key role as a regulator of the AR pathways in BC cell lines. MiR-9-5p was downregulated in the BC cell lines (T-47D, MDA-MB-453, and MCF-7). Transfection with miR-9-5p resulted in the significant downregulation of AR, both at the mRNA and protein levels. Interestingly, miR-9-5p is upregulated after androgen stimulation, which indicates a feedback loop between miR-9-5p and AR [45]. MiR-18a belongs to a miR-17-92a cluster and is significantly upregulated in BC [105,106]. Although the miR-17-92a cluster is upregulated by the AR, its exact role in BC progression remains unknown. As shown by Ottman et al., cluster miR-17-92a may induce prostate cell sensitivity to drugs (docetaxel, bicalutamide) and the AKT inhibitor MK-2206 2HCl. The inhibition of this cluster seems to be a promising therapeutic strategy [107]. Whether similar dependence also occurs in BC requires further investigation. Shi et al. have provided additional insight into the interactions of miRNAs and AR in BC. A comparison of the level of miRNA expression between AR-positive and AR-negative BC cell lines uncovered more than 150 differentially expressed miRNAs in AR-positive BC cells. The most significantly upregulated miRNAs were miR-933 and miR-5793, and the most downregulated was miR-4792. Many of the upregulated miRNAs were connected to BC cell proliferation, invasion, and drug resistance. Furthermore, networks of connection for predicted target genes showed their involvement in VEGF (vascular endothelial growth factor) and in the mammalian target of rapamycin (mTOR) signalling pathways, which are significant in BC tumorigenesis [46].

### 4.2. AR and miRNA in Prostate Cancer

The androgen receptor plays an important role in both the normal development of the prostate gland and its abnormal growth. The roles of AR in both miRNA regulation and miRNA-mediated regulation have mostly been extensively studied and documented in PCa studies. In 2007, Porkka et al. showed that androgens may regulate the expression of specific miRNAs in PCa cell lines [108]. Later, Epis et al. demonstrated that AR signalling was indirectly regulated by miR-331-3p in LNCaP cells [109]. In their studies, Stope et al. have shown that tumour suppressor miR-1 and small heat shock protein beta-1 (HSPB1) are involved in the development of PCa. The previously mentioned miR-21 is not only important in BC but also in PCa progression. Ribas and colleagues indicated that AR binds to miR-21 promoter and increases its expression, suggesting direct transcriptional regulation in PCa cell lines [99]. In PCa cells, HSPB1 reduces the expression of miR-1 and subsequently restores the oncogenic signalling pathways of AR. Interestingly, miR-1 overexpression significantly decreased PCa cell proliferation [110,111,112]. MiR-3162-5p has been shown to affect the proliferation and migration of PCa cells, while regulating kallikrein-related peptidase (*KLK*) and AR, by directly targeting their expression [113]. In contrast, AR may target AR/miR-4496/β-catenin signalling by regulating the expression of miR-4496 via direct binding to ARE sequences within the miR-4496 promoter [36]. More recently, miR-760 showed lowered expression in PCa tissues compared to in normal tissues. It was proven that androgens inhibit the expression of this miRNA in the LNCaP and 22rv1 cell lines. The downregulation of miR-760 promoted proliferation and growth in the PCa cell lines. Furthermore, miR-760 bound to the 3′UTR of interleukin-6 (IL-6) and inhibited its expression. This study demonstrated that androgens downregulate miR-760 to promote the growth of PCa cells by regulating IL-6 [47]. Another interesting study identified the key miRNAs in PCa using bioinformatic analysis [49]. The authors integrated gene expression and miRNA–mRNA association data to construct networks of hub miRNAs, and proposed candidate miRNA molecules that were specific to PCa occurrence and progression. Seven identified miRNAs (miR-1-3p, miR-125b-5p, miR-145-5p, miR-182-5p, miR-198, miR-24-3p, and miR-34a-5p) have been previously described to be involved in PCa and AR functioning, and two miRNAs, miR-22-3p and miR-499a-5p, have been proposed as candidates for new PCa biomarkers [114,115,116,117]. In addition, using bioinformatic and molecular analysis, Martinez-Gonzalez et al. indicated that miR-210-3p, miR-23c, miR-93-5p, and miR-592 were further potential non-invasive biomarkers for PCa. Interestingly, the implication of miR-23c in PCa has been shown for the first time [50]. Castration-resistant prostate cancer (CRPC) is a type of PCa that progresses despite medical or surgical castration [118]. In a profiling study, Rönnau et al. demonstrated several novel miRNAs that were significantly dysregulated in CRPC compared to in primary PCa tissue. MiR-3195, miR-3687, and miR-4417 were upregulated, while miR-205 and miR-92b were downregulated, in CRPC [51]. Interestingly, cell culture experiments showed a reduction in the expression levels of miR-3687 and miR-4417 in androgen-treated VCaP and LNCaP cell lines. MiR-1205 showed lowered expression in the PCa cell lines and tissues when compared to normal prostate epithelial cells and normal prostatic tissue. Furthermore, mice with CRPC treated with the miR-1205 synthetic analog (NB1205) showed smaller volumes of prostate tumour, which suggests that miR-1205 may have tumour suppressive properties in PCa. The FRY-like transcription coactivator (*FRYL*) is a direct molecular target for miR-1205, and is overexpressed in PCa tissue and in PCa cell line models. The overexpression of miR-1205 induces FRYL protein inhibition [52].

Circular RNA (circRNA) is an endogenous, non-coding, single-stranded RNA with a covalently closed loop with no 5′ cap or 3′ poly(A) tail [119,120]. Most recent studies indicate that the functioning of miRNA can be also regulated by circRNAs, which act as sponges and compete with mRNAs to bind to miRNAs [121,122]. Zhang et al., identified five circRNAs that were related to AR signalling and PCa progression [48]. Bioinformatic analysis proposed a network of over 200 possible interactions for these circRNAs with miRNAs, using the miRDB database for miRNA target prediction and functional annotations. MiR-216a-5p, miR-183-5p, and miR-206 were previously reported as being important components in PCa [123,124].

### 4.3. AR and miRNA in Other Genitourinary System Diseases

MiRNA expression is often altered not only in PCa, but also in other genitourinary cancers [125,126]. Furthermore, in these types of cancers, abnormal miRNA interactions with AR have also been described. Renal cell carcinoma (RCC) is the most common kidney cancer and is formed from renal tubular epithelial cells [127,128]. In 2017, Huang et al. indicated a unique mechanism by which AR increased or decreased clear cell RCC (ccRCC) metastasis. The majority of ccRCC subtypes lack the *VHL* gene (von Hippel–Lindau), which leads to the stabilization of hypoxia-inducible factors (*HIF*) and increased expression of vascular endothelium growth factors (VEGFs). Interestingly, AR-positive ccRCC cells preferably metastasize to the lung rather than to the lymph nodes. Moreover, AR may elevate the expression of miR-185-5p through binding to its AREs, which subsequently suppresses the expression of *VEGF-C* (vascular endothelial growth factor C) and increases the levels of *HIF2α/VEGF-A* (hypoxia-inducible factor 2 alfa/vascular endothelial growth factor A) [53]. In addition, AR has been shown to affect the metastasis of *VHL* wild-type clear cell RCC [54]. Depending on oxygen availability, AR transcriptionally suppresses or promotes miR-185-5p expression, resulting in changes in the *VEGF-C* and *HIF2α/VEGF-A* levels. A study by Wang et al. showed that AR may also increase the proliferation of RCC cells independently of the *VHL* status. Moreover, the authors found that the growth of RCC cells was promoted through the AR-dependent regulation of ASS1P3/miR-34a-5p/ASS1 signalling [55]. MiR-34a-5p was previously described as a tumour suppressor in RCC [129,130]. AR also negatively regulates the expression of miR-145, which leads to increased RCC cell proliferation and invasion [56]. Another interesting study showing the link between AR and miRNAs in RCC, indicated that AR effects ccRCC cell migration and invasion by changing circHIAT1/miR-195-5p/29a-3p/29c-3p/CDC42 signalling [57]. The suppression of circulating RNA circHIAT1 by AR resulted in altered miR-195-5p/29a-3p/29c-3p expression, which increased cell division cycle 42 protein (CDC42) expression, leading to intensified cell migration and invasion. Other studies have shown that AR may directly bind to the miR-143-3p promoter and potentially suppress its expression. Correspondingly, the novel long non-coding RNA lncRNA-SARCC (lncRNA-suppressing androgen receptor in renal cell carcinoma) has been shown to inhibit the AR protein by binding to it and destabilizing it, thus increasing the miR-143-3p level in RCC. The authors also found that Sunitinib (a protein tyrosine kinase inhibitor used in patients with RCC) induces lncRNA-SARCC expression, decreasing the resistance of RCC cells to this drug. These findings provide insight into the role of lncRNA-SARCC as a suppressor of RCC progression and highlight new therapeutic strategies for RCC treatment in the context of AR-miRNA regulation [58].

In bladder cancer, Yang et al. demonstrated that AR decreased the transcription of miR-525-5p by binding to different AREs located at different positions of the miR-525-5p precursor promoter, which subsequently altered miRNA-525-5p/SLPI (secretory leukocyte peptidase inhibitor) signalling and increased cancer metastasis [59]. An important link between miRNAs and AR involving lncRNA was presented by Xiong et al. LncRNA XIST (X-inactive specific transcript) promoted bladder cancer growth invasion and migration through the direct inhibition of miR-124, which is known to block AR expression by binding to the 3′UTR of AR [60].

In upper urinary tract urothelial cell carcinoma (UUTUC), the presence of AR in the CSC (cancer stem cell) population increased cell clonogenicity, in vitro spheroid formation, and changed the miRNA profile. Oncomirs miR-27a and miR-125b were upregulated in the BFTC hAR cells, while the tumour suppressors miR-145, miR-200b, and miR-200c were downregulated. This indicates that, in UUTUC cells, AR may upregulate the miRNAs that promote the expansion of the CSC population, and downregulate the miRNAs that suppress CSC population expansion. Further studies are required to understand the exact mechanism by which AR modulates CSCs by regulating miRNA networks. These findings provide new insight into AR functions in UUTUC [61].

At this point, it is worth mentioning that kidney stone disease is a common urological disorder [131]. Zhu et al. indicated that the loss of AR expression in renal tubular epithelial cells inhibits intrarenal calcium oxalate crystal deposition by altering macrophage recruitment and M2 macrophages polarization. Research suggests that AR can suppress macrophage colony-stimulating factor 1 (CSF-1) expression through the upregulation of miR-185-5p [132].

### 4.4. AR and miRNA in Liver Cancer

There is an increasing amount of evidence linking the AR to liver cancer [133,134]. Similarly, miRNAs, such as miR-216a, miR-155, or miR-21, have been implicated in the development of liver cancer [135]. Chen et al. showed that miR-216a and miR-224 were significantly upregulated in hepatocellular carcinoma (HCC), starting from the early stages of carcinogenesis. Interestingly, miR-216a levels were elevated in male patients, and the ligand-stimulated activation of AR led to the increased transcription of pri-miR-216a, indicating the possible involvement of androgens in the regulation of miR-216a biogenesis. It is important to note that this effect was not observed for miR-224 [62]. Another interesting study indicated a new AR/circ-LNPEP/miR-532-3p/RAB9A (AR/circ-leucyl and cystinyl aminopeptidase/miR-532-3p/ras-related protein rab-9a) signalling axis that is involved in the hypoxia-induced invasion of HCC cells. The researchers reported that the loss of AR under hypoxia increases HCC invasion via boost circ-LNPEP expression. This circular RNA acts as a sponge for miR-532-3p and consequently enhances the expression of RAB9A. This finding may help to provide new treatment strategies for HCC patients, which may include enhancing AR expression [63].

In a more recent paper, Huang et al. described the use of multiple omics integration in HCC research. The authors used miRanda software to predict the miRNA binding sites of the circRNAs within the junction regions, and collected mRNA–miRNA interactions from the miRTarBase database. A competing endogenous RNA network was then constructed using the miRNAs and differentially expressed genes, and the circRNAs indicated that some miRNAs (miR-6511a-5p and 4667-5p), as well as certain circRNAs (hsa_circ_0002130 and hsa_circ_0008774, hsa_circ_0008774), are connected to the AR in HCC [64].

### 4.5. AR and miRNA in Thyroid and Head and Neck Cancer

Stanley et al. reported a link between miR-124a and AR in thyroid cancer. In a target prediction analysis, *AR* was identified as a direct target of miR-124a. Experimental analysis confirmed that miR-124a determined the expression pattern of the *AR* gene in thyroid cancer tissues. In addition, miR-124a is considered to be a potential factor underlying the gender-specific expression of AR in thyroid cancer. The expression of AR mRNA was elevated in men and lowered in women (excluding follicular thyroid carcinoma) and showed a negative correlation with miR-124a expression. Interestingly, studies on cell lines have indicated that miR-124a diminishes cell proliferation [65].

In head and neck squamous cell carcinoma, a meta-analysis of miRNA expression combined with a protein–protein interaction network analysis identified over 50 differentially expressed miRNAs, where 16 miRNAs were involved in the regulation of AR. The analysis showed that some miRNAs are tumour-suppressing and that others play oncogenic roles. The overexpression of miR-7, miR-9, miR-15, miR-18, miR-19, miR-21, miR-23, miR-24, miR-93, miR-96, miR-99, miR-130, miR-139, miR-141, miR-155, miR-181, miR-195, miR-196, miR-210, miR-211, miR-214, miR-222, miR-296, miR-302, miR-331, miR-345, and miR-424 was associated with poor prognosis in head and neck squamous cell carcinoma. Decreased expressions of miR-17, miR-26, miR-29, miR-31, miR-34, miR-125, miR-126, miR-137, miR-138, miR-143, miR-152, miR-200, miR-203, miR-205, miR-206, miR-218, miR-324, miR-363, miR-375, miR-451, miR-489, miR-491, miR-506, miR-519, miR-639, and let-7d were correlated with lower survival and metastasis [66].

### 4.6. AR and miRNA in Pancreatic Cancer

Recent works have indicated the role of AR in the development of pancreatic cancer [136,137]. Rare solid-pseudopapillary neoplasms of the pancreas have been shown to have upregulated Wnt/β-catenin, Hedgehog, and AR signalling pathways, and miRNA profiling revealed that 17 miRNAs were associated with these pathways. Specifically, the AR could be targeted by miR-376b [67]. Another analysis of the miRNA profile in pancreatic cancer patients identified 10 miRNAs with the highest prognostic prediction values for pancreatic adenocarcinoma. A protein–protein interaction network analysis of the target genes for miR-376b and miR-376c indicated *AR* as a hub gene [68].

### 4.7. AR and miRNA in Lung Cancer

AR was found to be expressed not only in normal tissue, but also in cancerous tissues of the lungs [138,139]. Correspondingly, many studies emphasize the role of miRNAs as a potential biomarker for lung cancer [136,140]. In addition, Bouhaddioui et al. showed that the miRNAs involved in lung development in foetal mice were androgen-dependent [137]. In an interesting study by Jin et al., the authors analysed RNA sequencing data from blood samples taken from lung cancer patients and healthy controls. They identified 59 differentially expressed miRNAs. Transcriptional factor regulatory network analysis showed that the miR-657 target *WT1* (Wilms’ tumour gene) and the miR-582-5p target *ETV1* (ETS variant 1) regulate the *AR* gene at the same time [69]. These studies demonstrate the need for further research on AR–miRNA regulation in lung cancer.

### 4.8. AR and miRNA in Cardiovascular Diseases

Cardiovascular disease is still the leading cause of mortality worldwide [141]. There is substantial evidence regarding the relationships between AR and hypertension, stroke, atherosclerosis, and myocardial infarction [142]. However, knowledge of AR–miRNA interactions is still scarce. Research on mice with experimental autoimmune myocarditis has indicated that the AR regulates cardiac fibrosis by increasing the expression of miR-125b [143]. Idiopathic pulmonary artery hypertension, chronic thromboembolic pulmonary hypertension, and acute pulmonary embolism are serious pulmonary vascular diseases. Recently, through the use of small RNA sequencing, Fabro et al. identified several miRNAs that were distinctly dysregulated in these diseases. Importantly, for two of them, let-7i-5p and miR-320a, the *AR* was a target gene [70].

Chronic thromboembolic pulmonary hypertension (CTPH) is a potentially fatal disease that may occur as a rare complication following acute pulmonary embolism. The main cause of CTPH is a blockage in the blood vessels that may lead to heart failure [144]. Miao et al. identified miRNAs that are connected with CTPH [71]. MiR-3148, one of the key differentially expressed miRNAs, was downregulated in CTPH patients compared to in healthy donors. Importantly, the AR was found to be a target for miR-3148.

Shi et al. showed that hyperglycaemia triggered increased expression of miR-21-3p in cardiac fibroblasts. Interestingly, miR-21-3p repressed the expression of AR by binding to the 3′UTR of the *AR* gene. As a consequence, AR downregulation led to the pyroptosis of the cardiac fibroblasts and collagen decomposition through caspase-1 activation [145].

### 4.9. AR and miRNA in Ovarian Cancer and Polycystic Ovary Syndrome

It has been shown that androgen signalling plays an important role in tumorigenesis and metastasis in ovarian cancer [146,147,148]. MiRNA profiling of serum from patients who were either at high or low risk of ovarian cancer development, revealed almost 140 differentially expressed miRNAs. Most of them were downregulated in patients with a high risk of ovarian cancer, and in the targeted genes involved in hypoxia and androgen signalling. The most downregulated and validated miRNAs were miR-93-5p, -19a-3p, -22-3p, -362-5p, and -210-3p. The authors suggested that the alteration of miRNA levels may contribute to the upregulation of genes involved in the androgen signalling pathway, such as macrophage colony-stimulating factor (*CSF-1*), macrophage colony-stimulating factor receptor (*CSF-1R*), and Erb-B2 receptor tyrosine kinase 4 (*ErbB4*), during the initial stages of ovarian cancer [72]. AR–miRNA interactions have also been reported in polycystic ovarian syndrome (PCO). Murri et al. identified 38 differentially expressed miRNAs in the serum of patients with PCO. Moreover, a prediction of putative miRNA target genes showed that several of these miRNAs, such as miR-30c-5p, miR-34c-5p, miR-142-3p, miR-199a-3p, miR-224-3p, miR-548d-3p, miR-597-5p, miR-598-3p, miR-1468-5p, miR-107, miR-151a-3p, miR-199a-5p, and miR-153, may participate in AR signalling. Additionally, *AR* was predicted to be a target for miR-30c-5p, miR-199-5p, and miR-597 [73]. In another study, researchers investigated the miRNA levels in women with PCO (without insulin resistance) and estimated the free androgen index to determine androgen status. Importantly, studies have shown that the expression of miRNAs, such as miR-18b-5p, miR-424-5p, and let-7b-3p, although upregulated in PCO, does not correlate with androgen levels. Only for miR-1260a has a significant correlation with the free androgen index been demonstrated [74].

### 4.10. AR and miRNA in Trophoblast and Placenta Development

Shao et al. described a specific mechanism underlying the production of androgens and estrogens in the human placenta, which is regulated by miR-22 [75]. A higher miR-22 level was observed in early-onset preeclampsia placentas compared to unexplained preterm labour placentas. In human placental trophoblasts, testosterone blocked estradiol production by upregulating the miR-22 level [75]. Recently, McWhorter et al. reported on the connection between let-7c miRNA and AR in the trophoblasts. In the human first-trimester trophoblast cell line, a trophoblast differentiation-related RNA-binding LIN28B protein was shown to regulate AR expression via let-7c. The inhibition of LIN28 resulted in reduced AR expression, increased levels of let-7, and increased trophoblast differentiation [76]. These observations are crucial for understanding the mechanisms underlying abnormalities in trophoblast cell differentiation, which can lead to placental disorders, including preeclampsia.

### 4.11. AR and miRNA in Adipogenesis

The AR is present in preadipocytes, and adipocytes and androgens are involved in adipose tissue functions and fat distribution in the body [149]. Recently, the interactions of AR with several miRNAs have been described in human adipogenesis [150]. For example, miR-130a was significantly upregulated under androgen stimulation during the early phase of adipogenesis, and *AR* has been shown to be a target for miR-130a together with adiponectin (*ADIPOQ*) and tumour necrosis factor alpha (*TNFα*) [77]. During adipogenesis, another miRNA, miR-375, is also regulated by androgen signalling; this miRNA is upregulated during adipogenic differentiation and downregulated following androgen treatment [78]. Interestingly, miR-375 was previously described as being upregulated in PCa tissues [151], as an inhibitor of nasopharyngeal carcinoma cells [152], as well as being related to diabetes [153]. The adipose tissue regulates insulin sensitivity, and the AR may play a role with androgens in pancreatic islet β cells and diabetes [154]. Interestingly, a bioinformatic analysis of the key genes and molecular mechanisms involved in insulin resistance indicated that, among miRNAs that control differentially expressed genes in patients with subcutaneous insulin resistance, 96 miRNAs are involved in the downregulation of the AR, which was presented in the protein–protein interaction network [79]. This study presented a new potential relationship between miRNAs and the AR that is worth considering in further investigations into insulin resistance.

## 5. Is There a Potential for miRNA-Based Therapy in AR-Dependent Malignancies?

The above examples indicate a strong association between different miRNAs and AR. Although further studies involving large groups of patients are needed to validate the importance of these interactions, these works provide a hope for the development of new treatment strategies that target both AR and miRNAs. The ability of miRNAs to target multiple genes within a signalling pathway either in many types of cancer or in other diseases makes them very promising targets for the development of new therapeutic approaches. In general, miRNA-based therapeutics act as miRNA antagonists and mimics [155]. MiRNA mimics are chemically modified, double-stranded RNAs that can imitate endogenous mature miRNAs and restore their functionality [156]. MiRNA antagonists are single-stranded, antisense oligonucleotides that can be applied to intercept and degrade mature miRNAs [157]. The biggest challenge in this kind of therapy is deriving an efficient delivery system and acquiring an exact understanding of miRNA function in a specific disease. The delivery of synthetic miRNAs may be achieved through a few strategies, including oligonucleotides with chemical modifications, liposomes, polymers, hydrogels, nanoparticles, lentiviruses, and adenoviruses [18]. For example, Devulapally et al. presented a model in which polymer nanoparticles could successfully deliver anti-miR-10b and anti-miR-21 in TNBC to block apoptosis and metastasis [158]. Exosomes have been shown to effectively deliver anti-miR-21 in PCa, allowing miR-21 downregulation and the blockade of cancer progression in the PC-3 human prostate cancer cell line [159]. Successful miR-155 and miR-124 delivery through the use of nanoparticles has also been described in ovarian cancer [160,161]. Montgomery and colleagues applied cholesterol conjugates to successfully deliver the miRNA-29 mimic to mouse lung tissue, which restored miRNA-29 function, decreased collagen expression, and repressed pulmonary fibrosis [162]. MiRNAs are not only targets for new therapies, but may also serve as prognostic biomarkers for various diseases. The indisputable advantage of using miRNAs as biomarkers is that miRNAs can be collected and detected in biofluids, such as serum, plasma, blood, tears, urine, or saliva, in a minimally invasive way [163,164,165]. Additionally, these small particles are stable, remaining so long after collection. The limitation of their usage is that the miRNA level required could be dependent on age, gender, and previously applied treatments [166,167].

Regardless of the current limitations in the delivery of synthetic miRNAs to cells, one can expect that the combined use of miRNA with standard chemotherapy targeting AR may improve the outcome in future patients. Androgen deprivation therapy (ADT) is the first-line treatment used for patients with PCa [168]. Lowering androgen levels or blocking AR binding to testosterone prevents AR activation, and at least temporarily blocks PCa progression [169]. 

Combinations of different therapies with standard ADT are highly investigated. Javed et al. provide a summary of AR signalling in a normal and an aggressive form of PCa, and its relationships with miRNA and curcumin in potential therapeutic approaches. Curcumin is a compound of natural origin that has antioxidant, anti-inflammatory, and anti-cancer properties like preventing metastasis or limiting cancer cell proliferation [170,171]. MiRNAs, such as miR-34a, miR-143, miR-770-5p, miR-1247, and miR-145, showed elevated expression in the curcumin-treated PCa cell lines, and as a result, halted migration or cell proliferation was observed [172]. Fletcher et al. showed that the inhibitors of miR-346, miR-361-3p, and miR-197 were found to reduce the transcriptional activity of the AR, mRNA, and protein levels, and to significantly inhibit migration and invasion in the PCa cell culture. The inhibition of these miRNAs presents additive effects with antiandrogens that might be promising for combination approaches in PCa treatment [173]. In their study, Lin et al. used PCa and healthy prostate cell lines to examine the relationship between miR-31 and AR. It was discovered that miR-31 targets *AR*, and that its upregulation inhibits the expression of the AR at the protein and RNA levels, which suppresses PCa development. MiRNA-31 inhibits AR expression by binding to the coding region of the AR mRNA. Furthermore, the AR can suppress miR-31. These findings might help to design a therapy that supports existing therapies that focus on blocking AR activity [174]. It has been proven that miR-133a-5p targets fused in sarcoma (FUS) proteins and the AR in PCa cell lines. The overexpression of miR-133a-5p significantly downregulated FUS and AR, and suppressed the cell proliferation of the AR-positive PCa cell lines. Inversely, the inhibition of miR-133a-5p enhanced the expression of FUS and AR, and consequently PCa cell proliferation [175]. Lyu et al. presented how DHT significantly upregulates let-7a expression and inhibits cell proliferation in the ER-, PR-, and AR+ BC cell lines. The inhibition of let-7a expression by antisense oligonucleotides revealed the upregulation of the MYC proto-oncogene (CMYC) and KRAS proto-oncogene (KRAS) protein, and elevated BC cell growth [176]. Recent results indicate that AR is able to suppress the formation of a new HCC vascularization pattern (vasculogenic mimicry) by regulating the circRNA7/miR-7-5p/VE-cadherin/Notch4 (circulating RNA 7/miR7-5p/vascular endothelial cadherin/notch receptor 4) signalling axis in HCC cell lines. The overexpression of AR upregulates miR-7-5p expression through the inhibition of circRNA7 in HCC cell lines. MiR-7-5p directly targets *VE-cadherin* and *Notch4* and decreases their expression, which inhibits vasculogenic mimicry formation. To block this abnormal vascularization, the pattern inhibition of circRNA7 expression, as well as miR-7-5p expression recovery, could be effective [177].

The above-mentioned studies showing the relationships between AR and miRNAs in various diseases provide useful information for developing new therapies. Epigenetic therapies can aid and enhance the effect of existing approaches. 

## 6. Conclusions

A growing amount of evidence is produced each year regarding the importance of the interactions that take place between miRNA and the AR. Although relationships between the miRNAs and the AR are predominantly studied in relation to prostate cancer and breast cancer, the role of miRNAs and the AR in other diseases has begun to emerge. The knowledge of the molecular mechanisms governing the mutual regulation of the AR and miRNAs will undoubtedly help us to design better therapeutic strategies and to provide more accurate molecular diagnostic, prognostic, and predictive biomarkers for specific diseases.

## Figures and Tables

**Table 1:** The main new findings about miRNA–AR relation in different conditions in humans.

Condition	miRNA	Significant Findings and Implications Concerning AR–miRNA Interactions	Year of Publication	Reference
Breast cancer	miR-185, miR-205, miR-21	Disturbance of AR, miR-205, miR-185, and miR-21 expression may be the marker for the presence of metastases depends on the tumour subtype.	2020	[40]
	miR-21	AR downregulates miR-21 expression	2016	[41]
	miR-100, miR-125	AR regulates the extracellular release of MMP13 via the regulation of miR-100 and miR-125	2017	[42]
	miR-328-3p	DHT regulates miR-328-3p expression via AR	2018	[43]
		DHT controls chemo-response independently of ABCG2 and miR-328-3p	2021	[44]
	miR-9-5p	miR-9-5p acts as a tumour suppressor and downregulates AR	2020	[45]
	153 DE miRNAs in AR- positive BC including miR-933, miR-5793, miR-4792	miRNAs promote AR-mediated signalling BC progression	2017	[46]
Prostate cancer	miR-760	Downregulation of miR-760 promotes cancer cell growth by regulating IL-6	2021	[47]
	miR-216a-5p, miR-183-5p, miR-206, miR-3160-5p, miRNA-204-5p	Target prediction analysis for 5 circRNAs related to the AR signalling pathway showed circRNA—miRNA regulatory network with more than 200 interactions	2021	[48]
	miR-1-3p, miR-125b-5p, miR-145-5p, miR-182-5p, miR-198, miR-24-3p, miR-34a-5p, miR-22-3p, miR-499a-5p	miR-145-5p/NDRG2/AR and miR-145-5p/KLF5/AR axis were found to be potential mechanisms in PCa development	2021	[49]
	miR-210-3p, miR-23c, miR-592, miR-93-5	miR-210-3p, miR-23c, miR-592, and miR-93-5 as a potential diagnostic and aggressiveness biomarkers for PCa	2021	[50]
	miR-3195, miR-3687, miR-4417	Upregulation of miR-3195, miR-3687, and miR-4417 in PCa	2021	[51]
	miR-1205	miR-1205 act as a tumour suppressor through the regulation of FRYL	2021	[52]
Renal cell carcinoma	miR-185-5p	AR elevates the expression of miR-185-5p, which suppresses VEGF-C and increases HIF2α/VEGF-A expression	2017	[53]
		AR affects RCC metastasis via regulation of miR-185-5p	2020	[54]
	miR-34a-5p	AR increases proliferation of RCC cells through regulation of ASS1P3/miR-34a-5p/ASS1 signalling	2019	[55]
	miR-145	AR negatively regulates miR-145, which enhances RCC cell invasion and proliferation	2015	[56]
	miR-195-5p, 29a-3p, 29c-3p	AR promotes RCC cell migration and invasion by regulating circHIAT1/miR-195-5p/29a-3p/29c-3p/CDC42 signals	2017	[57]
	miR-143-3p	lncRNA-SARCC suppresses RCC progression via altering AR/miRNA-143-3p signalling	2017	[58]
Bladder cancer	miR-525-5p	AR binds to different AREs on the miR-525-5p promoter region and increases metastasis in bladder cancer	2020	[59]
	miR-124	XIST inhibits miR-124 expression; miR-124 regulates AR expression	2017	[60]
Urothelial carcinoma	miR-27a, miR-125b, miR-145, miR-200b, miR200c	AR promote expansion of CSC	2016	[61]
Liver cancer	miR-216a, miR-224	miR-216a and miR-224 are upregulated in HCC tissues	2012	[62]
	miR-532-3p	AR/circ-LNPEP/miR-532-3p/RAB9A signalling axis may be committed to hypoxia-induced cell invasion of HCC cells	2021	[63]
	miR-6511a-5p, miR-4667-5p	Competing endogenous RNA network analysis indicates that some miRNAs and circRNAs are connected to AR in HCC	2021	[64]
Thyroid cancer	miR-124a	AR is a target for miR-124a; miR-124a determines the expression of AR gene in human thyroid cancer tissues	2012	[65]
Head and neck cancer	53 DE miRNAs	A total of 16 miRNAs might be involved in the regulation of AR in head and neck cancer	2017	[66]
Pancreatic cancer	232 DE miRNAs including the miR-200 family and miR-192/215	AR is targeted by miR-376b	2014	[67]
	494 miRNAs	PPI network analysis of target genes for miR-376b and miR-376c showed AR as a hub gene	2018	[68]
Lung cancer	59 DE miRNAs	Transcriptional factor regulatory network showed miR-657 as regulator of AR expression	2017	[69]
IPAH, CTEPH, APTE	21 DE miRNAs including let-7i-5p, miR-320a miR-320b-1, miR-320b-2, miR-1291	AR is a target gene for let-7i-5p and miR-320a	2021	[70]
CTEPH	46 DE miRNAs including miR-3148	AR is a target for miR-3148	2017	[71]
Ovarian cancer	137 DE miRNAs including miR-93-5p, miR-19a-3p, miR-22-3p, miR-362-5p, miR-210-3p	Most of tested miRNA target genes were connected to hypoxia and androgen pathways	2019	[72]
PCO	38 DE miRNAs included miR-30c-5p, miR-34c-5p, miR-142-3p, miR-199a-3p, miR-224-3p, miR-548d-3p, miR-597-5p, miR-598-3p, miR-1468-5p, miR-107, miR-151a-3p, miR-199a-5p, miR-1539	AR is a target of miR-30c-5p, miR-199-5p, and miR-597; other miRNAs possibly involved in AR signalling	2018	[73]
	miR-1260a, miR-18b-5p, miR-424-5p, and miR-let-7b-3p	miR-1260a corelate with androgen levels	2020	[74]
Early-onset preeclampsia placentas	miR-22	Production of androgen and estrogen is modulated by miR-22	2017	[75]
Placenta development	let-7c	LIN28 regulates AR expression via let-7c	2019	[76]
Adipogenesis	miR-130a, miR-301	miR-130a is upregulated under androgen stimulation in the adipogenesis; *AR* is a target gene for miR-130a	2020	[77]
	miR-375	miR-375 is upregulated during adipogenic differentiation and is downregulated after androgen treatment	2015	[78]
Insulin resistance	miRNA profile	PPI network indicated that AR was regulated by 96 different miRNAs in subcutaneous insulin resistance	2019	[79]

ABCG2: ATP-binding cassette subfamily G member 2; APTE: acute pulmonary embolism; BC: breast cancer; AR: androgen receptor; ARE: androgen response element; circRNA: circular RNA;; CSC: cancer stem cells; CTEPH: chronic thromboembolic pulmonary hypertension; DE: differentially expressed; DHT: dihydrotestosterone; HCC: hepatocellular carcinoma; HIF2α: hypoxia-inducible factor 2 alfa; IL-6: interleukin 6; IPAH: idiopathic pulmonary artery hypertension; MMP13: metalloprotease-13; PCa: prostate cancer; PPI: protein-protein interaction;f;on; RCC: renal cell carcinoma; VEGF-A: vascular endothelial growth factor A; VEGF-C: vascular endothelial growth factor C.

**Table 2:** Key oncomiRs and suppressor miRNAs connected with androgen receptors in different types of cancer.

Cancer Type	miRNA	miRNA Type	Reference
Breast cancer	miR-185	suppresor	[40]
	miR-21	oncomiR	[41]
	miR-100	suppresor	[80]
	miR-328-3p	suppresor	[44]
	miR-9-5p	suppresor	[45]
Prostate cancer	miR-760	suppresor	[47]
	miR-204-5p	suppresor	[48]
	miR-34a-5p, miR-145-5p	suppresor	[49]
	miR-93-5	oncomiR	[50,81]
	miR-1205	suppresor	[52]
Renal cell carcinoma	miR-185-5p	oncomiR	[53]
	miR-34a-5p	suppresor/oncomiR	[55]
	miR-145	suppresor	[56,82]
	miR-195-5p, 29a-3p, 29c-3p	suppresor	[57]
	miR-143-3p	suppresor	[58]
Bladder cancer	miR-525-5p	suppresor	[59]
	miR-124	suppresor	[60,83]
Urothelial carcinoma	miR-27a, miR-125b	oncomiR	[61]
	miR-145, miR-200b, miR-200c	suppresor	[61]
Liver cancer	miR-216a	oncomiR	[62]
	miR-532-3p	oncomiR	[63,84]
Thyroid cancer	miR-124a	suppresor	[65,85]
Head and neck cancer	miR-100	suppresor	[66,86]
Pancreatic cancer	miR-200 family	suppresor	[67,87]
Lung cancer	miR-1197	oncomiR	[88]
Ovarian cancer	miR-93-5p	suppresor	[72,89]
